# Social care causes of delayed transfer of care (DTOC) from hospital for older people: Unpicking the nuances of ‘provider capacity’ and ‘patient choice’

**DOI:** 10.1111/hsc.13911

**Published:** 2022-07-16

**Authors:** Kate Gridley, Kate Baxter, Yvonne Birks, Louise Newbould, Stephen Allan, Daniel Roland, Gintare Malisauskaite, Karen Jones

**Affiliations:** ^1^ Social Policy Research Unit University of York York UK; ^2^ PSSRU University of Kent Canterbury UK

**Keywords:** delayed transfers of care, discharge from hospital, older people, qualitative research, social care, social care market

## Abstract

Unnecessarily prolonged stays in hospitals can have negative impacts on patients and present avoidable costs to health and social care systems. This paper presents the qualitative findings of a multi‐methods study of the social care causes of delayed transfers of care (DTOC) for older people in England. The quantitative strand of this study found that DTOC are significantly affected by homecare supply. In this paper, we explore in depth how and why social care capacity factors lead to delays, from the perspectives of those working within the system. We examined the local transfer arrangements in six English local authority (LA) sites that were purposively sampled to include a range of DTOC performance and LA characteristics. Between March and December 2018, 52 professionals involved in arranging or facilitating discharge from hospitals in these sites provided qualitative data, primarily through semi‐structured interviews. Topics included discharge teams and processes, strategic issues and perceived causes of delays. The thematic analysis uncovered the nuances behind the causes of DTOC previously categorised broadly as ‘provider capacity’ and ‘patient choice’. In particular, our analysis highlights the lack of fit between available provision and the needs of people leaving hospital (theme 1); workforce inconsistencies (theme 2) and a myth of patient choice (theme 3). We are now at a turning point in the development of policy to reduce DTOC in the English system, with the full implications of a new national discharge to assess programme yet to be seen. Our research shows the significance of the alignment of service capacity, including the type and location of provision, with the needs and preferences of those leaving hospital. As the new system becomes established, attendance to such nuances behind blockages in the system will be more important than ever.

**What is known about this topic**

- Delays in hospital discharge can have a detrimental impact on individuals, families and systems
- A majority of delayed transfers of care in England involve older patients
- Little is known about the nuances behind the headline causes of ‘provider capacity’ and ‘patient choice’ to which social care‐related delayed discharges are often attributed

**What this paper adds**

- In poor‐performing localities, professionals involved in discharge identified a lack of fit between available provision and the needs of older people leaving hospital
- Workforce inconsistencies can lead to chains of delay
- Delays attributed to ‘patient choice’ may in part reflect a lack of acceptable options for patients and families to choose from

## 1 INTRODUCTION

Older people are admitted to hospital when the medical imperative to admit outweighs the importance of remaining in a familiar setting, with familiar routines and opportunities for mental and physical activity. When the immediate medical need is met, however, transferring the patient out of hospital becomes the priority. It is now recognised that ‘Unnecessarily prolonged stays in hospital are bad for patients’ (NHS Improvement, 2018, p. 2) and there is substantial evidence to suggest that hospital stays can have detrimental effects on the health of older people in particular (Brown et al., 2009; Covinsky et al., 2003; Jasinarachchi et al., 2009; Kortebein et al., 2008; Rojas‐García et al., 2018).

Delayed transfers of care reduce the number of beds available for emergency and planned admissions. Internationally, there is evidence this leads to avoidable costs (Holmås et al., 2013; Micallef et al., 2022; Rojas‐Garcia et al., 2018). In England, a National Audit Office (2016) survey of hospitals suggested that 85% of delayed transfers of care involved patients aged 65 or over with estimated additional National Health Service (NHS) costs of around £820 million per annum. On top of this, there are additional costs to social care, as prolonged inactivity can lead to reduced functional ability and increased needs for care services. Data are limited, but the National Audit Office estimates additional costs to the community health and social care system of around £180 million per annum (National Audit Office, 2016).

There is growing interest worldwide in transitions from hospital and the impact of delays (Allen et al., 2017; Forchuk et al., 2019; Kosteniuk et al., 2021) with a recent international review flagging the importance of understanding the context within which initiatives to reduce delays operate (Cadel et al., 2021). In England, partially in response to the need to free up hospital capacity during the Covid‐19 pandemic (Chartered Society of Physiotherapy, 2020), a national discharge to assess programme was implemented in 2020. In this new system, only limited assessment is carried out in hospital, with the focus instead on comprehensive home assessments and up to 6 weeks of funded support in the home. For those whose needs are too great to return home (approximately 5% of patients over 65), short‐term care and rehabilitation in a residential facility will be arranged (HM Government, 2020, 2021).

As part of these developments, service providers are currently not required to record the Delayed Transfer of Care (DTOC) data which were the linchpin of discharge policy and target setting in England (HM Government, 2020). Previously, health and social care organisations were required to count and report delays via a national system (Unify) and give a reason for each, including whether the delay was caused primarily by health or social services (NHS England, 2018). This system was suspended on 19th March 2020. We are now, therefore, at a turning point in the development of policy to reduce delayed discharges in the English system and as such it seems salient to take stock and ensure that learning from previous approaches is not lost. While each country's health and social care system is different, insights from developments in England can be translated into learning for other countries.

### 1.1 The English context

The English National Health Service (NHS) is publicly funded and generally free to use, with hospital services in an area usually provided by one or two acute trusts. Social care, on the other hand, is means tested and provided by multiple independent (private or not for profit) providers employing their own care workers and other support staff (Quilter‐Pinner & Hochlaf, 2019). Social care in this context typically refers to the provision of ‘packages’ of care (support with washing, dressing and other activities of daily living) for people in their own homes, or residential care provided by care or nursing homes. However, social care is complex, encompassing multiple other services including aids and home adaptations, daycare and support for family carers. Local authorities (LAs) in each area have a responsibility to work with local providers to ensure that social care needs are met (Local Government Association, 2018).

The Unify system provided detailed data on the numbers and causes of DTOC from hospital to home or another setting in England. These data tell us that DTOCs increased considerably between 2014 and 2017, peaking at an average of 6660 beds per day in February 2017 (NHS England, 2019). A gradual reduction since then coincided with a national policy drive requiring LAs to agree targets for reducing DTOCs caused by social care and risk losing funding if they performed poorly (NHS England, 2017). The percentage of delays attributed to social care has consistently been lower than that attributed to health. Nevertheless, delays where the person was awaiting a care package into their own home made up the largest percentage of all delays (20.8%) in 2018/2019 (NHS England, 2019).

An early systematic review of delayed discharges of older people from hospital in England (Glasby et al., 2006) identified a number of social care‐related causes including awaiting care home placement/availability, awaiting domiciliary/community package/lack of community services, staff shortages and housing/aids and adaptations/social circumstances. These issues recurred frequently in the literature over the following decade (Baumann et al., 2007; Bryan, 2010; Hendy et al., 2012; Jasinarachchi et al., 2009). However, most studies tended simply to list the general service areas accountable for delays (e.g. ‘combined social and therapy delay’ or ‘lack of downstream bed’ in Hendy et al., 2012) or general issues (e.g. ‘seeking of care home placement’ or ‘family delays’ in Bryan, 2010).

More recent research has shown that older patients with specific characteristics tend to be delayed more than others, for example, those with both cognitive impairment and physical dependency (Challis et al., 2014), poorer mobility prior to admission and confusion at the time of admission (Jasinarachchi et al., 2009). Patients in old age psychiatry wards with greater cognitive impairment have also been shown to be more likely to be delayed than those with less cognitive impairment (Tucker et al., 2017).

In this paper, we present the qualitative findings of a multi‐methods study on DTOC (Jones et al., 2019). The quantitative element found that DTOCs were significantly affected by homecare supply, with every extra homecare provider per 10 km^2^ decreasing DTOCs attributed to social care by 14.9%, equivalent to 449 days per year for the average LA (Allan et al., 2021). These findings, however, tell us little about the *type* of provision or the nature of capacity required to reduce DTOCs. This paper complements the quantitative findings by exploring in detail how and why capacity factors lead to delays, from the perspectives of those working within the system.

## 2 METHODS

We employed a multiple case studies approach (Yin, 2011), aiming to explore, in detail, the local transfer arrangements in six LA case sites, primarily using in‐depth interviews with health and social care professionals involved in facilitating discharge. We aimed to carry out up to eight interviews in each site covering details of teams and processes, strategic issues, perceived causes of delays and facilitators of smooth transfers. Key contacts in each site identified further stakeholders to invite to interview. Interviews were conducted by one of two researchers (KG and KB) at a location and time convenient to the participant and generally lasted between 30 and 60 min. Focus groups were offered as an alternative means of participating. All participants received an information sheet and gave informed consent prior to participating. The interviews and focus group were audio‐recorded with participants' permission and fully transcribed. Health Research Authority approval was granted in February 2018 (IRAS project ID: 243467).

### 2.1 Recruitment of sites and participants

Sites were purposively sampled to include a range of DTOC performance and characteristics (rural/urban, deprivation, ethnic diversity and level of apparent integration of services) to allow comparative analysis and identification of contextual factors contributing to delays (see Table 1). Sites 1 and 2 had seen a recent positive improvement in DTOCs (based on our team's analysis of DTOC between 2010 and 2016 which controlled for LA characteristics: Jones et al., 2019). Sites 3 and 4 had seen recent deterioration in DTOCs (based on this same analysis). Sites 5 and 6 were selected based on their absolute DTOC days, which for Site 5 were more than double the national average (from last quarter 2010 to last quarter 2013, continuing to rise until 2015) and for Site 6 were slightly above average until the end of 2013, but then increased sharply from the first quarter 2014 to the last quarter 2016.

**TABLE 1:** Overview of case study site characteristics, DTOCs and participants

Site	Description	DTOCs	Participants
Site 1	Rural Not deprived^a^ Over 90% White British Integrated hospital discharge team	Positive improvement in DTOCs in 2016 compared to 2010–2016^c^	11 participants: Hospital Discharge Team Manager, Safeguarding Team Manager, Long‐Term Team Manager, In‐reach Nurse, Care Manager and Assistant Care Manager, 2x Social Workers, Senior Physiotherapist, Occupational Therapist and OT Assistant
Site 2	Urban Not deprived Less than 50% White British Integrated hospital discharge team	Positive improvement in DTOCs in 2016 compared to 2010–2016	9 participants: Director for Adult Social Care, Discharge Team Manager, Performance Analyst (Discharge), Discharge Team Manager and Deputy Manager, Discharge Team Safeguarding Lead, Mental Health Social Care Services Manager, Long‐Term Conditions Support Worker (voluntary sector), Service Manager (voluntary sector)
Site 3	Urban High levels of deprivation^b^ Over 80% White British Separate hospitals and LA discharge teams	Large negative change in DTOCs in 2016 compared to 2010–2016	6 participants: Community Manager ‐ Hospitals and Reablement (LA), Care Placement Team Worker (LA), Bed Utilisation Administrator (hospital), Hospital Social Work Team Leader, Community Manager ‐ Mental Health (LA), Case Management Team Manager (hospital)
Site 4	Urban Not deprived Over 80% White British Separate hospital and LA discharge teams	Large negative change in DTOCs in 2016 compared to 2010–2016	8 participants: Service Manager (LA), Discharge Planning Manager (LA), Service Manager (LA), Transfer of Care Team Lead (hospital), Deputy Chief Operating Officer (hospital), Mental Health Liaison Team Manager (care trust), Deputy Director of Nursing (care trust), Head of Commissioning Adult Social Care
Site 5	Urban Not deprived Over 80% White British Integrated hospital discharge team	DTOCs consistently above the national average from 2010 to 2016	10 participants: Commissioning Manager (dementia), 2x Care Home Managers, Social Work Team Manager, Hospital Team Manager (LA), Chief Officer for Access and Care (LA), Head of Specialist Services (LA), Integrated Discharge Team Manager, Clinical Service Manager (discharge), Carer Support Worker (voluntary sector)
Site 6	Mixed rural/urban Not deprived Over 80% White British Integrated hospital discharge team	DTOCs above average from 2010–2013, then increased sharply 2014 to 2016	8 participants: Assistant Director Care Pathway (LA), Head Adult Community Services (LA), Head of Service ‐ Adult Social Care, Director of Health and Care Integration Performance Management, Head of Nursing for Patient Flow and Discharge, Head of Patient Flow, Housing and Community Services Group Manager, Head of service Mental Health for Older People

a Does not feature in the 20 LAs with the highest proportion of their neighbourhoods in the most deprived 10% of neighbourhoods (Dept for Communities and Local Government, 2015) The English Indices of Deprivation 2015 https://www.gov.uk/government/collections/english‐indices‐of‐deprivation.

b 45% of its neighbourhoods were in the most deprived 10% of neighbourhoods nationally on the Index of Multiple Deprivation 2015.

c Jones et al. (2019).

Between March and December 2018, 52 professionals involved in arranging or facilitating discharge from hospital provided qualitative data for the case studies, primarily through face‐to‐face and telephone interviews (Table 1). Site 1 opted for a focus group as their primary mode of participation, supplemented by interviews (nine stakeholders attended the initial focus group and three follow‐up interviews were held, two of which were with additional participants).

### 2.2 Analysis

Data were analysed thematically (Miles & Huberman, 1994) by three researchers (KG, KB and LN) using the Framework approach (Ritchie & Lewis, 2003) within NVivo: a software package which enables transcripts to be coded (in this case, inductively) and contains an embedded matrix suitable for Framework analysis. Construction of the overarching framework had four stages: familiarisation with the data, identification of initial codes, full coding of all transcripts and summarisation of selected coded data into a code‐by‐case/participant chart. The causes of delay most commonly discussed by participants were Domiciliary care capacity, Residential/nursing home capacity and Patient and family ‘choice’. Text coded under these headings were entered into a higher level ‘central chart’ and analysed thematically focussing on the themes (or conceptually founded patterns, Braun et al., 2019) running through the text, and any differences between these in better and worse performing LAs. Case summaries were also developed and regularly consulted to ensure that site contexts remained central to our interpretation.

## 3 FINDINGS

Through this iterative process of moving between the case contexts and the code content, we reflexively developed three key themes associated with delayed discharge from hospital for older people, all of which speak to the nuances behind the headline explanations of limited provider capacity and patient choice. Theme 1 highlights the lack of fit between available provision and the needs of people leaving hospital. Theme 2 addresses issues related to the social care provider workforce. Theme 3 introduces the myth of ‘patient choice’, whereby delays attributed to people ‘choosing’ to stay in hospital may on closer inspection be caused by insufficient or mismatched supply limiting their options.

### 3.1 Theme 1: Lack of fit: the mismatch between available support and the needs of people leaving hospital

An inability to source packages of home care to meet the needs of people returning home from hospital was seen as a major cause of delays, especially in poor‐performing areas. At a surface level, problems in sourcing timely homecare packages were referred to in terms of limited care provider capacity. However, as interviewees gave more details about the circumstances of delays, it became clear that the absolute quantity of care providers was only part of the picture, with the complexity of the needs of discharged patients featuring highly in explanations. That is, the *type* of package required and how swiftly this could be operationalised were critical factors.

Packages of home care for people who would need two care workers to visit at the same time (known as ‘double‐up’ or ‘double‐handed’ packages), for instance, were harder to source in some areas than single‐handed packages. It was generally felt that the demand for more labour‐intensive packages had for some time been increasing as people's needs were becoming more complex: for example, growing numbers of bariatric service users requiring double‐handed packages. The number of people requiring multiple visits a day was also felt to be increasing:

So it's not just about social care having the space of ten patients, it might be that they've got the space of ten patients who need two calls a day, but actually we're discharging to them ten patients that need three or four calls a day … So it's volume and complexity that we're struggling with. (Site4_P5)

‘Time critical’ packages could also be problematic. These were for people who needed support at specific times of the day, for example to administer medication. Not only did this introduce inflexibility into the specification (a provider would need to have a care worker available *at the same time and location* every day) but multiple‐time critical packages tended to have similar requirements (medication to be administered at 9 am, for example) and this created pinch points of high demand that were sometimes difficult to meet even when there was slack in the system at other times.

Lack of capacity in the residential and nursing care markets was another cause of delays, especially for people leaving hospital with complex needs. Delays were repeatedly attributed to a mismatch between the types of placements available in the system and the requirements of people being discharged. A major cause of delays in Site 5, for example, was the lack of nursing placements for people with complex needs and ‘challenging behaviour’. In this area, there was an ample supply of residential beds, but if a person was assessed as presenting behaviour considered to be challenging (e.g. linked to dementia), some participants felt residential homes were reluctant to take them, meaning a nursing bed, in much shorter supply, was required. The availability of a bed in itself was therefore not enough for a person to be discharged; it had to be *the right kind of bed*. Staff‐to‐resident ratios also needed to be maintained, so while some homes could in theory take people with complex needs, in practice, they may not be able to if, for example, a number of their existing residents exhibited behaviour considered to be ‘challenging’ at that time. In addition to people with dementia, it could also be harder to find placements for people with some mobility problems, as these too required additional staffing to support them.

Similar issues were identified across all four poorly performing sites. In Sites 3 and 6, the same lack of nursing beds was felt to cause delays, especially for people with dementia. In Site 5, it was perceived that care homes cherry‐picked residents with less complex needs, leaving a small cohort of people that no provider would take and who were consequently subject to lengthy delays. Here, the lack of suitable placements was seen to be potentially linked to LAs offering a single ‘standard rate’ per resident to care homes, regardless of need:

…if we're paying care homes a standard rate then why would they take anyone more complex when they can take someone less complex? We've got a payment system that incentivises care homes not to take complex people. (Site5_P1)

In Site 4, a shortage of residential care providers able to care for people with complex needs meant those providers accepting such residents could charge a premium. Where the NHS covered the costs (through a scheme, called continuing healthcare, for people with very high health‐related needs), the additional time taken to approve these premium prices contributed to delays. Where premium prices were passed on to residents, this could also lead to delays if an individual or family would not (or felt they could not) pay extra. Thus, people could be waiting for a care or nursing home placement not strictly because of a lack of places but because of a lack of *affordable* places.

In an attempt to avoid delays linked to the complexity of residents' needs, the local commissioning body in Site 5 had begun to make additional short‐term funding available to care homes to, for example, pay for one‐to‐one staffing until a resident with complex needs was settled. They had also commissioned a new team whose role was to support care homes to manage risk and implement personalised interventions intended to help providers feel more confident to take residents with complex needs. However, these initiatives had only recently been established and it was not yet known what impact they would have on DTOCs.

### 3.2 Theme 2: Chains of delay and workforce issues

The impact of limited capacity reverberated throughout the system, delaying patients ready to be discharged directly from hospitals and also slowing discharges from reablement services (which provide support at home to help people achieve goals on discharge from hospital, similar to ‘restorative care’ in the USA and Australia) and other transitional services, which in turn meant less capacity for these services to take new patients from the acute sector:

…we've got patients who are on the reablement service that are waiting for an ongoing package of care; we've got patients who are in the neighbourhood teams who are receiving therapy, who have reached their therapy goals, who now need a long‐term package of care. So everybody's got patients who are going to the same bottleneck of waiting for packages of care… (Site5_P9).

By far, the most common complicating factor identified when sourcing new homecare packages, other than the complexity of the service user's needs, was their address. All of the low‐performing sites had locations within their boundaries for which there was little cover from any provider, even when capacity overall seemed adequate. These tended to be rural areas, wealthy areas or both. Site 2 provides a useful comparison. This was an entirely urban area with low and improving DTOC rates which did not report problems setting up packages of care in specific locations within its boundaries. However, this site was served by a discharge team which also covered a neighbouring LA with pockets of rurality. Managers covering both sites attributed the causes of delays to provider capacity in those rural areas, explaining they had the same discharge systems covering both locations, but in the rural authority, it could take days to source double‐handed packages, which in the urban site were arranged without delay.

Some interviewees felt that virtually full employment in wealthy (especially wealthy rural) areas made it challenging to make the care industry an attractive employer. Where care workers could not be recruited locally, they would have to travel from less wealthy areas and, for remote rural localities, this additional travel time reduced overall availability. In Site 5, a higher ‘rural rate’ was introduced to compensate providers for the additional travel expense, but they still reported problems sourcing packages in particular locations.

Problems with staff retention could exacerbate patchy coverage, especially at certain times of the year, such as school holidays (‘…because they're earning less than the childcare would be’. Site5_P6) and the run up to Christmas, when potential care workers might ‘…earn more working in Marks & Spencer's…’ (Site4_P5). Where this coincided with increased demand for health services over winter, the squeeze on capacity came from both sides, as hospitals attempted to discharge more patients (and patients themselves wished to go home for Christmas) just as competition from the Christmas retail sector meant recruitment and retention of care workers became more difficult. Competition for staff from other sectors that paid similar rates and had arguably better working conditions was a problem raised in all four of the poor‐performing sites.

Only a handful of homecare providers offered night‐time care. In Site 3, the LA had attempted to recruit personal assistants to provide night cover but, despite offering higher than usual pay, they were unable to recruit. Other timing issues, such as requests for care packages made towards the end of the week, were also problematic in some areas as providers did not always have the capacity to activate new care packages over the weekend.

The combination of factors relating to the type of support required, its timing and location meant that, even when capacity overall was adequate, delays could still be experienced by people wishing to return to particular locations:

…health might be saying they want four calls a day, probably double‐ups, four times two [workers] a day, and we're in disagreement with that, or we can't actually physically source that level of provision in some areas … they might be rural … the agencies can't get employees… (Site6_P1).

This participant advocated taking a more granular approach to unpick the detail of these challenges at a local level and link more closely with providers. Another participant felt that as the complexity of people's needs increased, the market needed to adapt to meet those needs. However, they also pointed out that there would need to be an incentive for the market to respond and questioned whether this existed at the present time, when there seemed to be more than enough work to keep providers in business.

### 3.3 Theme 3: Attributing delays to ‘patient choice’ can mask the scarcity of acceptable options

… sometimes we'll explore stuff like short‐term residential placements, to get that person off that acute ward before that care's in place, but often people just want to go home, they don't, older people especially they're very frightened of going into care, and families can be very resistant to that, which you can totally understand. So often, there can be quite a delay… (Site3_P4).

Under the Unify system, areas were required to report both the numbers of patients whose transfer of care was delayed and the reasons for these delays using pre‐set categories. Category G ‘Patient or Family choice’ indicated a delay when a patient had been made a ‘reasonable offer of services’ (a short‐term care home placement, e.g.) but had refused to accept this (NHS England, 2015, p. 12). Patient choice was identified (explicitly or implicitly) as a key cause of delays in all four of the poor‐performing sites. In Site 6, the number of days officially attributed to lack of homecare capacity was low (despite provider capacity being identified as an important cause of delays by interviewees) because when a homecare package was not available, a patient would be offered an interim bed instead. This would be counted as a ‘reasonable offer of services’ and if the patient turned the offer down the resultant delay would be logged as ‘G. Patient or Family choice’. The situation in Site 5 was very similar. If a package of care could not be sourced the person would be offered 4 weeks in a transitional bed (usually in a residential home) funded by the LA, and sometimes the patient would turn this down:

A: …the highest cause of delay is G‐code…

Q: Which is?

A: Which is family choice, patient and family choice. Because what we do is if somebody's waiting for a care package, and it might be a week, 10 days, it's 11 days average to get a care package so it can be longer, what we do is we'll offer the person a stay in an alternative provision, so we've got step‐up step‐down beds that we offer to people….

Q: But they might choose not to take them?

A: Exactly. If they choose not to we would then put them on a G‐code because we have offered a suitable alternative. (Site5_P6).

Participants here talked about the drawbacks of this approach, particularly the apparent contradiction between the policy aspiration to prioritise getting patients home (NHS England, 2018) and the reality of offering someone a residential place when they were medically fit to return to their own home with a care package. In Site 3, the response to lack of homecare capacity was similarly to offer patients a temporary bed. Here they flagged the risk that in doing so you could ‘miss your window to get them home’ (Site3_P5), especially for people with dementia where additional moves could exacerbate dementia symptoms.

A common reason for people refusing a residential placement (either temporary or longer term) was the geographical location of the home. If people were offered a place in a home that was hard for family to reach or that was unfamiliar to the person, this could be refused and would be logged under the patient choice code. Similarly, if people chose a conveniently located home that had no vacancies and insisted on waiting for a vacancy to become available, the resultant delay would be categorised as patient choice (rather than lack of provider capacity in the desired location). Sometimes, people hoped a longer stay in hospital would mean sufficient recovery to move to a residential rather than a nursing home. Options were constrained further when preferences about type and location of home combined. In one such case, a family ruled out several care homes, while several others (which they preferred) said they could not meet the person's needs. As a result, this person had been delayed in hospital for 6 months awaiting discharge. In some sites, there had been initiatives to remind and encourage families and patients that once they were medically fit they would be expected to leave hospital, but at the same time, some recognition that lack of availability limited patients' choices. As a participant in Site 4 explained

…obviously we are working with people, not tins of beans. So, you know, there's all those intricacies to be fed in about “well I don't want to be in a home on the north side of the city”… (Site4_P2).

Some of these issues were also reported in Site 2, one of the high‐performing sites, which might indicate that delays attributable to ‘patient and family choice’ cannot be designed out of the system. Nevertheless, it was clear that all four poor‐performing sites were experiencing a mismatch between the type and location of supply and the wants and needs of patients and families.

## 4 DISCUSSION

This study illustrates a complex picture of uneven supply and multifaceted demand that goes beyond the traditional headline that ‘lack of social care capacity’ and ‘patient choice’ lead to delays. We found that the lack of fit between available provisions and the needs of people leaving hospital, combined with workforce pressures in certain locations and at certain times, contributed to a system that consistently served some patients and localities better than others. Meanwhile, this picture had been distorted by a reporting system that attributed a significant proportion of delays to the individual choices made by patients and families, when on closer inspection those choices were often constrained by limited capacity to meet their needs and preferences.

The quantitative findings of our wider study showed a clear relationship between DTOCs and the number of homecare providers in a geographical area (Allan et al., 2021) and there is evidence that the number of care home places in a locality also influences DTOC and length of stay (Fernandez et al., 2018; Gaughan et al., 2015). Our interviews with the professionals involved in sourcing and setting up homecare packages illustrated the fine‐grained nuances of the capacity issues faced, which were as often down to the types of packages required, the times of day and the locations in which they were required, as the total numbers of providers available. Similarly, with residential and nursing care, the absolute number of beds alone did not always determine provider capacity; rather the type of homes available, the mix of residents and the combination of their needs against providers' staffing levels and expertise also affected their ability to take on new residents.

Delayed discharge from hospital is a problem internationally, but evidence suggests interventions to accelerate discharge through structured discharge planning have only a minimal impact on length of stay (Gonçalves‐Bradley et al., 2022). Such interventions tend to focus on improving systems within the hospital setting. What they do not typically tackle is the nature or capacity of the care system patients are discharged into. Similarly, international commentators have consistently identified a ‘lack of flow’ between acute and community services, but the focus has tended to be on communication and coordination rather than the nature of supply and demand (Dossa et al., 2012; Leclair et al., 2021; Sheehan et al., 2021). Studies identifying issues with homecare provision often neglect to set out precisely what the nature of these problems are (Bragstad et al., 2012; Bryan, 2010; Hendy et al., 2012). Workforce instability is one factor known to affect care supply internationally, with low pay and low status making the care sector an unattractive employer, especially in higher‐income countries (Shinan‐Altman et al., 2020; Stone, 2017). In England, local authorities have a responsibility to work with local providers to develop a shared understanding of current and future social care needs and ensure these can be met (Local Government Association, 2018), but the stakeholders we spoke to identified mismatched supply and demand linked to problems recruiting and retaining care workers in the places and at the times they were required.

Another factor affecting systems the world over is the increasingly complex needs of those leaving hospital (Kuluski et al., 2017; Lenzi et al., 2014). One of our case study areas had introduced capacity building and financial support to address some of the issues affecting providers' ability to support individuals with complex needs and counter the disincentive of paying a standard rate for all residents. There is mixed evidence on the effects of adult social care expenditure on healthcare utilisation, including DTOC (Crawford et al., 2018; Iparraguirre, 2020; Liu et al., 2021; Seamer et al., 2019). Our study suggests a direct link between low LA standard rates paid for care home placements and higher delayed discharges, particularly for people with complex needs. Conversely, we also found that delays could be caused when providers charged a premium price (in keeping with Gaughan et al., 2015). It may be that the factors delaying some people (e.g. those seeking affordable care to meet ‘standard’ needs) might be quite different from the issues facing others (such as those seeking specialist support for dementia or other complex conditions). This further illustrates the importance of attending to the nuances at the intersection of provider capacity, cost and type and complexity of needs in order to fully understand the causes of delays.

Patient choice has been identified as a major cause of delays in England (NHS England, 2015) and internationally (Tan et al., 2010). Our analysis echoes that of Cornes et al. (2008) who questioned whether the choice of discharge destination is always a genuine choice. Stakeholders highlighted to us that people's preferred choices were not always available or not in locations that were suitable. When choices are limited or unsatisfactory, it is perhaps not surprising that some people will ‘choose’ not to take what is offered, especially when they are funding the care themselves. This issue may have even greater relevance now that a new national system of discharge to assess has been introduced across England, which includes the routine transfer of patients whose needs are too great to return home immediately to short‐term residential/rehabilitation care (HM Government, 2020). Again, the situation is nuanced. While delays in discharge may have emotional and physical costs for patients and families, there is also evidence to suggest that rushing discharge to free up beds can cause worry and dissatisfaction and potentially influence readmission rates (Cornes et al., 2008; Friebel et al., 2019; Fuji et al., 2013; Rojas‐García et al., 2018). The stakeholders we interviewed highlighted the fear and resistance often present when older people were faced with the option of short‐term residential care when their preference was to return straight home or move directly to a longer‐term residence of choice. The new hospital discharge guidance states that ‘If another care setting is required, the end point is to get people home as soon and as safely as possible.’ (HM Government, 2020, p10). However, there is evidence that the relocation of older people with dementia can negatively impact their physical and mental well‐being (Ryman et al., 2019) and the stakeholders we interviewed raised the concern that for some people, especially those with dementia, multiple moves could reduce the chances of them ever returning home. It is not yet clear how disagreements between patients/families and discharge teams will be tackled under the new system, but it will be important to monitor this and remain cognizant of the possible links between delays attributed to apparent ‘choice’ and provider capacity that meets patients' needs and preferences.

### 4.1 Strengths and limitations

While we included a range of case study sites with varied success in terms of DTOC performance, the sites were to some degree self‐selected. Three of the six LAs initially approached to take part declined. It is impossible to say whether those three sites had common characteristics or experiences that discouraged them from participating and may have also been relevant to the research. We were, nevertheless, able to include the perspectives of a wide range of stakeholders, identified as key players in their local systems, ensuring that within sites, we reflected a breadth and depth of experience not often captured qualitatively (Cadel et al., 2021).

## 5 CONCLUSION

This research shows the importance of paying attention to the specific nature of available provision in specific locations against the needs and preferences of those leaving hospital. As the new discharge to assess system becomes more established in England, attendance to nuances behind blockages in the system will be more important than ever.

## AUTHOR CONTRIBUTION

All listed authors have contributed to the manuscript substantially and have agreed to the final submitted version.

## ACKNOWLEDGEMENTS

We are grateful to all those who gave their time and expertise to participate in this study, and to those who helped to recruit case study sites ad participants.

## FUNDING INFORMATION

The study represents independent research funded by the National Institute for Health Research (NIHR) School for Social Care Research (NIHR SSCR). The views expressed are those of the authors and not necessarily those of the NIHR SSCR, NIHR or Department of Health and Social Care.

## CONFLICT OF INTEREST

The authors declare that they have no conflict of interest to disclose.

## Data Availability

The authors elect to not share data (consent was given by research participants for their data to be used only for this specific study).
